# Low-cost flexible supercapacitors with high-energy density based on nanostructured MnO_2_ and Fe_2_O_3_ thin films directly fabricated onto stainless steel

**DOI:** 10.1038/srep12454

**Published:** 2015-07-24

**Authors:** Girish S. Gund, Deepak P. Dubal, Nilesh R. Chodankar, Jun Y. Cho, Pedro Gomez-Romero, Chan Park, Chandrakant D. Lokhande

**Affiliations:** 1 Thin Film Physics Laboratory, Department of Physics, Shivaji University, Kolhapur, - 416004 (M.S), India; 2 Department of Materials Science and Engineering, Seoul National University, Seoul 151-744, South Korea; 3 Catalan Institute of Nanoscience and Nanotechnology, CIN2, ICN2 (CSIC-ICN), Campus UAB, E-08193 Bellaterra (Barcelona), Spain

## Abstract

The facile and economical electrochemical and successive ionic layer adsorption and reaction (SILAR) methods have been employed in order to prepare manganese oxide (MnO_2_) and iron oxide (Fe_2_O_3_) thin films, respectively with the fine optimized nanostructures on highly flexible stainless steel sheet. The symmetric and asymmetric flexible-solid-state supercapacitors (FSS-SCs) of nanostructured (nanosheets for MnO_2_ and nanoparticles for Fe_2_O_3_) electrodes with Na_2_SO_4_/Carboxymethyl cellulose (CMC) gel as a separator and electrolyte were assembled. MnO_2_ as positive and negative electrodes were used to fabricate symmetric SC, while the asymmetric SC was assembled by employing MnO_2_ as positive and Fe_2_O_3_ as negative electrode. Furthermore, the electrochemical features of symmetric and asymmetric SCs are systematically investigated. The results verify that the fabricated symmetric and asymmetric FSS-SCs present excellent reversibility (within the voltage window of 0–1 V and 0–2 V, respectively) and good cycling stability (83 and 91%, respectively for 3000 of CV cycles). Additionally, the asymmetric SC shows maximum specific capacitance of 92 Fg^−1^, about 2-fold of higher energy density (41.8 Wh kg^−1^) than symmetric SC and excellent mechanical flexibility. Furthermore, the “real-life” demonstration of fabricated SCs to the panel of SUK confirms that asymmetric SC has 2-fold higher energy density compare to symmetric SC.

Energy storage devices are undergoing an intense scientific and technological evolution in order to adapt to a new sustainable energy model that will need to foster renewable power and autonomous electric vehicles. Both batteries and supercapacitors are benefitting from intense efforts to overcome their major drawbacks, that is, slow rates and low power for batteries and relatively low energy density in the case of supercapacitors^1^. But in addition to improved intrinsic performance of electrode materials there is a need to explore and exploit alternative designs for the fabrication of low-cost and new added value devices^2^.

Thus, in addition to increasing the energy density of supercapacitors through the use of pseudocapacitive or hybrid materials^2^,^3^, the introduction of cost-effective materials and processes based on safer and environmentally friendly technologies should be an important goal. Furthermore there is the issue of new added value devices. For example, multifunctional and portable electronic appliances, like wearable electronics, smart sensors and actuators, miniature biomedical devices, smart garments, flexible touch screens and electronic newspapers, demand light weight, flexible and highly integrated electronic devices to be folded, rolled up, or even remolded into other structures easily and safely. Flexible-solid-state supercapacitors (FSS-SCs) are the only choice for meeting all of the above demands being also safer (both non-toxic and less reactive) and longer-lasting than batteries^4^,^5^,^6^.

Among improvable aspects, the energy density of SC devices is probably the number one challenge. Enhancing energy density can be accomplished by increasing the specific capacitance (C_s_) and/or the operation voltage (V) of the cell (since E = 1/2 CV^2^)^7^. In order to follow the former approach many different transition metal oxides (MO), carbon allotropes (fullerenes, carbon nanotubes, graphene and activated carbons) and their composite materials have been tested in order to take advantage of their pseudocapacitance while enhancing electrochemical performances through improving specific surface area, porosity and conductivity^8^,^9^. Recently, nanoporous metal oxides and carbons have been synthesized using metal-organic frameworks (MOF) as a template, which can render highly nanocrystalline and advanced specific surface area material leads to better transportation and adsorption of chemical agents^10^,^11^,^12^. On the other hand, in order to boost up specific energy by increasing the working voltage, two main routes could be followed: i) the use of organic electrolytes in symmetric SCs and ii) the design of asymmetric SCs^7^. The use of organic solvents in the electrolyte presents several intrinsic problems (lower conductivity, reduced safety and environmental friendliness). Thus, the proper design of asymmetric SCs with eco-friendly technology and low-cost fabrication emerges as the primary way to enhance energy density by increasing voltage.

There are several possible combinations of metal oxide (MO) and carbon materials (C) in the design of asymmetric SCs, namely, MO//C^13^,^14^,^15^,^16^, MO//MO^17^,^18^,^19^,^20^, MO/C//C^7^,^21^,^22^ and MO/C//MO/C^23^,^24^. Among MO//MO designs the system combining MnO_2_ as the positive electrode and Fe_2_O_3_ as the negative electrode in combination with a solid electrolyte, is especially enticing when compared with others. Indeed, these oxides are non-toxic and eco-friendly materials which can be extracted from very abundant low-cost minerals through simple syntheses. Previously, Cottineau *et al.*^25^, and Brousse *et al.*^18^ combined MnO_2_ as a positive electrode and Fe_2_O_3_ as a negative electrode, preparing bulk powders of MnO_2_ and Fe_2_O_3_ by chemical methods and painting them on stainless steel substrates, obtaining specific capacitances (energy densities) of 21.5 F g^−1^ (17.3 W h kg^−1^) and 20 F g^−1^ (7 W h kg^−1^), respectively (with a working potential of 1.8 V in aqueous 0.1 M K_2_SO_4_ electrolyte). Recently, Yang *et al.*^20^ also combined the same electrode materials, prepared by chemical methods on carbon fabric for the fabrication of asymmetric FSS-SC device and got specific capacitance (energy density) of 91.3 F g^−1^ (33.5 W h kg^−1^) with 1.6 V potential. However, neither of those configurations is optimal for the design of thin, flexible or wearable energy storage devices. Liquid electrolyte in the two former reports and commercial carbon fabric as a substrate in the latter, point to their use in more conventional, bulk supercapacitors. Thus, our efforts focused on getting a unique combination of optimal elements for the low-cost fabrication of thin and flexible MnO_2_-Fe_2_O_3_ supercapacitors, including cheap fabrication technology, use of polymer gel electrolyte, highly flexible stainless steel (SS) substrate and use of simple chemical methods (facile and eco-friendly routes allowing for the synthesis of large area electrodes). Some of the methods reported for the synthesis of MO electrodes (MnO_2_ and Fe_2_O_3_) in thin film form include electrodeposition^26^,^27^,^28^, chemical bath deposition^29^,^30^,^31^, sol-gel^32^,^33^, successive ionic layer adsorption and reaction (SILAR) method^34^,^35^ etc. All of them with specific advantages and drawbacks.

We report here the fabrication and testing of thin flexible solid state supercapacitors (FSS-SCs) based on MnO_2_ (electrodeposited) and Fe_2_O_3_ (SILAR method) electrodes, which allowed for the direct deposition in thin film form through facile, fast, cost-effective, additive and binder-free, and eco-friendly synthesis routes suitable to prepare large area electrodes. A neutral gel (1 M Na_2_SO_4_/Carboxymethyl cellulose (CMC)) was used as electrolyte. Symmetric (MnO_2_/MnO_2_) and asymmetric (MnO_2_//Fe_2_O_3_) FSS-SC devices were demonstrated and tested.

## Results

### Fabrication of the MO electrodes

The primary objective of the work is the scalable preparation of high-performance MnO_2_ and Fe_2_O_3_ electrodes for their integration in FSS-SC devices. Potentiodynamic (cyclic voltammetry) electrodeposition and SILAR methods were employed to synthesize MnO_2_ and Fe_2_O3 electrodes, respectively. Various MnO_2_ samples were prepared using scan rates of 50, 100 and 200 mV s^−1^ (samples denoted MnO50, MnO100 and MnO200, respectively). The SILAR synthesis of Fe_2_O_3_ was carried out at temperatures of 313, 333 and 353 K (samples labeled FO40, FO60 and FO80, respectively). Uniform and smooth porous structures with nano-channels suitable for easy impregnation of electrolyte and ion diffusion were obtained. MnO50 and FO60 samples exhibited the best electrochemical performance (See supporting information S1, S2, S4 and S5 for detail discussion of preparation, morphological, structural and electrochemical results of all electrodes). Thus, only these samples were considered and chosen for further discussion and use as optimized electrodes (hereafter denoted MnO_2_ and Fe_2_O_3_). The mass of active material for MnO_2_ and Fe_2_O_3_ electrodes was 0.18 and 0.26 mg cm^−2^, respectively.

**Figure 1:**
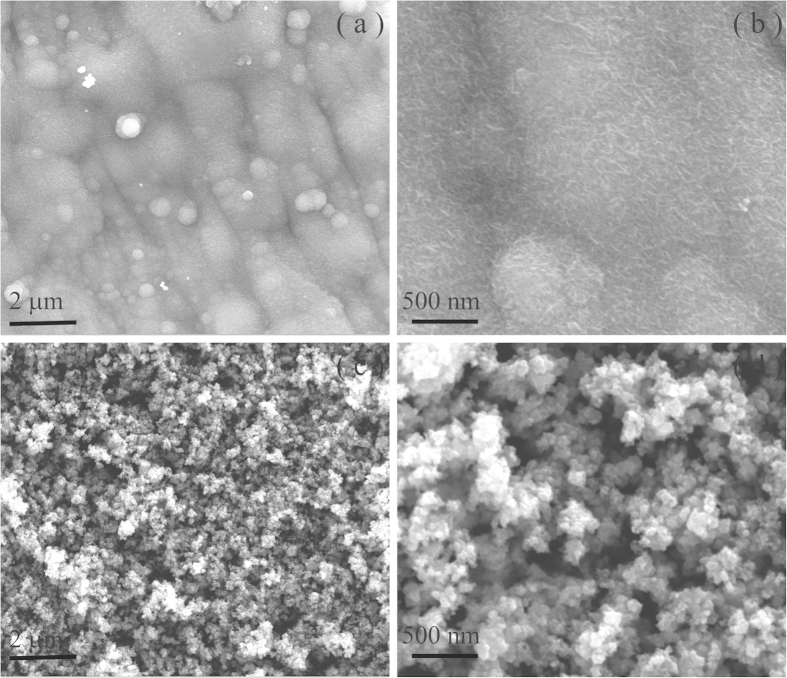
FE-SEM images of (**a**,**b**) MnO_2_ NSs and (**c**,**d**) Fe_2_O_3_ NPs electrodes.

The surface morphology of the as-prepared MnO_2_ and Fe_2_O_3_ electrodes on highly flexible SS sheet are shown in the SEM micrographs of Fig. 1(a–d), respectively. As shown in Fig. 1(a,b), MnO_2_ electrode shows a thin microporous structure consisting of very fine nanosheets (NSs) ca. ~15 nm thick. All these nanosheets are interconnected to each other and vertically aligned on the SS sheet. The scanning electron microscope (SEM) image of the Fe_2_O_3_ electrode displays uniform covering of highly porous microstructure composed of fine interconnected nanoparticles (NPs) about ~20 nm of diameter on SS sheet (Fig. 1(c,d)). These porous microstructures can foster ion transport and diffusion through the electrodes.

**Figure 2:**
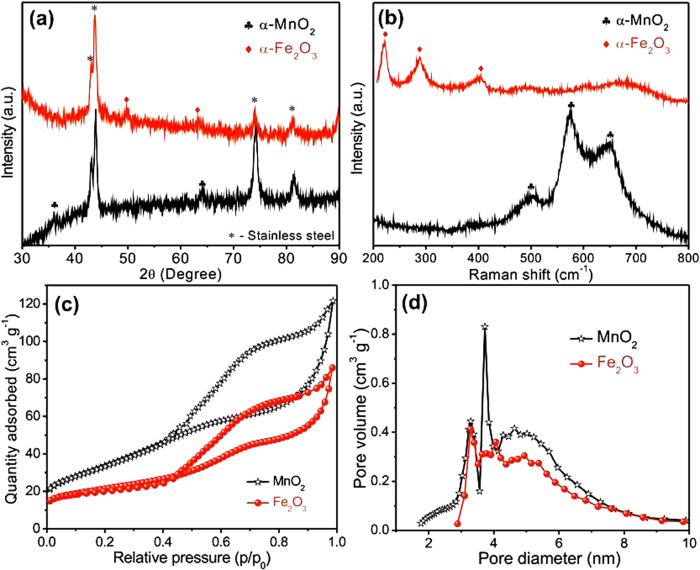
(**a**) XRD patterns (**b**) Raman spectra of MnO_2_ and Fe_2_O_3_ electrodes. (**c**) Nitrogen adsorption-desorption isotherms and (**d**) pore size distribution curves of powder MnO_2_ and Fe_2_O_3_ samples.

The structural investigation of the as-prepared MnO_2_ and Fe_2_O_3_ electrodes on SS sheet were carried out through X-ray diffraction (XRD) analysis (Fig. 2(a)). The peaks marked with an asterisk are associated to the X-ray reflections from the SS substrate. The two characteristic peaks, for MnO_2_ electrodes and planes are found at 37° (400) and 64.5° (002) (denoted by clubs) and can be indexed matching the α- phase of MnO_2_ (JCPDS No. 44-0141). In the case of Fe_2_O_3_ electrodes diffraction peaks at 50° (024) and 63° (300) (marked as diamonds) are associated to the α-phase of Fe_2_O_3_ (JCPDS No. 06-0502)^36^. All the peaks are weak and broad, which is related to the thin deposits and to the poor crystallinity of electrode materials, respectively. Such a poor crystallinity is usually associated to a low lattice energy, which can improve the utilization ratio of electrode materials by allowing for an easy de-intercalation process^7^.

In order to support the conclusions from XRD measurements, Raman spectroscopy studies were carried out (Fig. 2(b)), since each of the possible phases of MnO_2_ and Fe_2_O_3_ has a unique Raman spectrum. Thus, in this case, Raman spectroscopy is a best suited tool to confirm particular phases of MnO_2_ and Fe_2_O_3_. The Raman spectrum of the MnO_2_ deposit shows three peaks centered at 501, 575 and 651 cm^−1^ distinctive of α-MnO_2_ phase^37^. The spectrum of Fe_2_O_3_ shows peaks at 221, 288 and 403 cm^−1^ characteristic of α-Fe_2_O_3_^38^.

In order to quantify the porosity of the electrodes N_2_ adsorption−desorption isotherms were measured for powder samples of the MnO_2_ and Fe_2_O_3_ materials scratched from the corresponding electrodes (Fig. 2(c,d)). The appearing adsorption-desorption isotherms are distinguished and graphic of an isotherm curves for highly porous surfaces. Both MnO_2_ and Fe_2_O_3_ showed Brunauer−Deming−Deming−Teller (BDDT) data with the characteristic behavior of type IV isotherms with a H3-type hysteresis loop in the IUPAC classification, indicating the presence of micropores and mesopores with specific surface areas of 122.4 and 85.4 m^2^ g^−1^, respectively. The pore size distribution of the fine interconnected NSs and NPs nanostructure was fitted via Barrett−Joyner−Halenda (BJH) model (Fig. 2(d)). The resulting plots clearly show that there is major volume of pores in the micro and mesopore ranges. The micropores are the consequence of micro-slits formation due to interconnected NSs (MnO_2_) and NPs (Fe_2_O_3_) and are likely beneficial for the easy transport and diffusion of electrolyte ions.

### Electrochemical supercapacitive performance of MO electrodes

**Figure 3:**
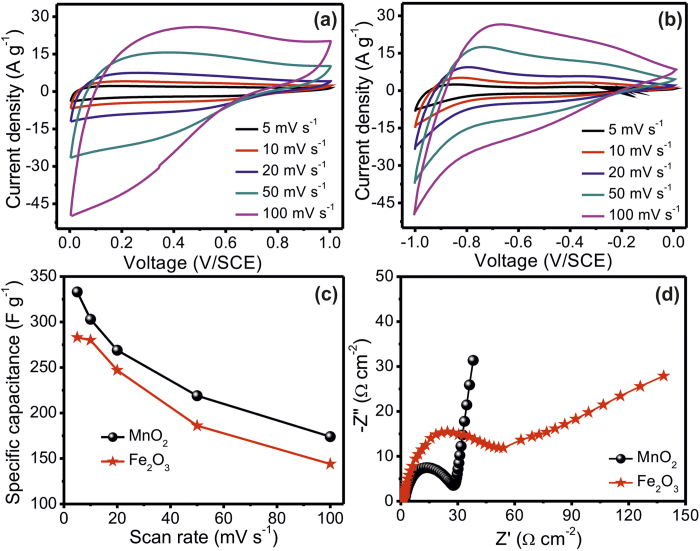
Cyclic voltammograms of (**a**) MnO_2_ and (**b**) Fe_2_O_3_ electrodes at different scan rates, (**c**) plots of specific capacitance versus potential scan rate. (**d**) Nyquist plots of MnO_2_ and Fe_2_O_3_ electrodes.

We performed cyclic voltammetry (CV) and electrochemical impedance measurements for MnO_2_ and Fe_2_O_3_ electrodes, in order to investigate their capacitive behavior, electrochemical stability and mechanistic ion transport properties, before employing these electrodes for device fabrication. The CV studies of interconnected NSs (MnO_2_: positive electrode) and NPs (Fe_2_O_3_: negative electrode) were carried out in 1 M Na_2_SO_4_ electrolyte within 0 to +1 and −1 to 0 V/SCE operational windows, respectively at different scan rates (5 to 100 mV s^−1^) using a three-electrode cell configuration, see Fig. 3(a,b). The cell consists of interconnected NSs and NPs electrodes as a working electrode (1 cm^2^), platinum as a counter electrode and saturated calomel electrode (SCE) as reference electrode. The enhancement of current density with increasing scan rate, for both NSs containing MnO_2_ and NPs covered Fe_2_O_3_ electrodes, indicates excellent supercapacitive behavior for both electrodes. The non-rectangular shape of the CV profile is associated with the faradic reactions i.e. reduction (M^x^ to M^x−1^) and oxidation (M^x−1^ to M^x^) on the electrodes^39^. The estimated specific capacitances and potential dependent charge storage (i.e. capacitive retention relating to scan rate) of the positive and negative electrodes through CV analysis are portrayed in Fig. 3(c). The estimated values of specific capacitance for positive and negative electrodes are 333 and 283 F g^−1^, respectively at 5 mV s^−1^ scan rate. The depletion of charge storage capacity (from 333 to174 F g^−1^ for positive and 283 to 144 F g^−1^ for negative electrode) with the potential is clearly displayed from Fig. 3(c) (the capacitive retention with respect to scan rate exhibited in supporting information 5(a)), which is associated to ion exchange mechanism. Particularly, highly porous nanostructures of MO electrodes need enough time for intercalation-deintercalation process during charging-discharging, so most of the electrode material remains unused at high potential due to fast rate of intercalation and deintercalation processes^40^. A good electrochemical stability of the electrodes is a must for their practical application in SC fabrication. Accordingly, CV measurements of positive and negative electrodes were repeated for 3,000 cycles at the scan rate of 100 mV s^−1^ and the calculated capacitive retention as a function of cycles for both electrodes displayed in supporting information 5(b). The capacitive remnants for positive and negative electrodes after 3,000 CV cycles were determined to be 91 and 75%, respectively. The better capacitive retention of positive electrode as compare to negative electrode may be consequence of good adhesion and less dissolution of MnO_2_ NSs as compare to Fe_2_O_3_ interconnected NPs in electrolyte during the cycling. Furthermore, the ion transport features of the prepared electrodes were examined through electrochemical impedance spectroscopy (EIS) analysis with AC amplitude of 10 mV and in a frequency range of 100 mHz −100 kHz. The obtained Nyquist plot (Z” vs Z’) comprises a high frequency semicircle and low frequency straight line, as illustrated in Fig. 3(d). The intercept of high frequency semicircle on real axis is associated with the equivalent series resistance (R_s_) (combination of ionic resistance of electrolyte, intrinsic resistance of electrode material and interfacial resistances), while the radius of the semicircle offers information on the charge transfer resistance (R_ct_) (consequence of Faradaic and non-Faradaic reactions on the electrode surface)^41^,^42^. The slope of the straight line in the low frequency region indicates the capacitive behavior of the electrode: reflection of excellent capacitive behavior associated to inclination from 45° to 90° with the fall of frequency. The observed values of R_s_ and R_ct_ indicate excellent ion conductivity of positive (1.4 and 1.2 Ω cm^−2^, respectively) and negative (14.8 and 28.6 Ω cm^−2^, respectively) electrodes on SS sheets; see Fig. 3(d). The slightly better performance of positive electrode (i.e. charge storage capacity, electrochemical stability and conductivity) is associated to the highly porous nanostructure and different mass loading on electrodes. In any event, the excellent electrochemical properties of the chemically prepared MnO_2_ and Fe_2_O_3_ electrodes point to their efficient utilization for device fabrication.

### Symmetric and Asymmetric Supercapacitors

**Figure 4:**
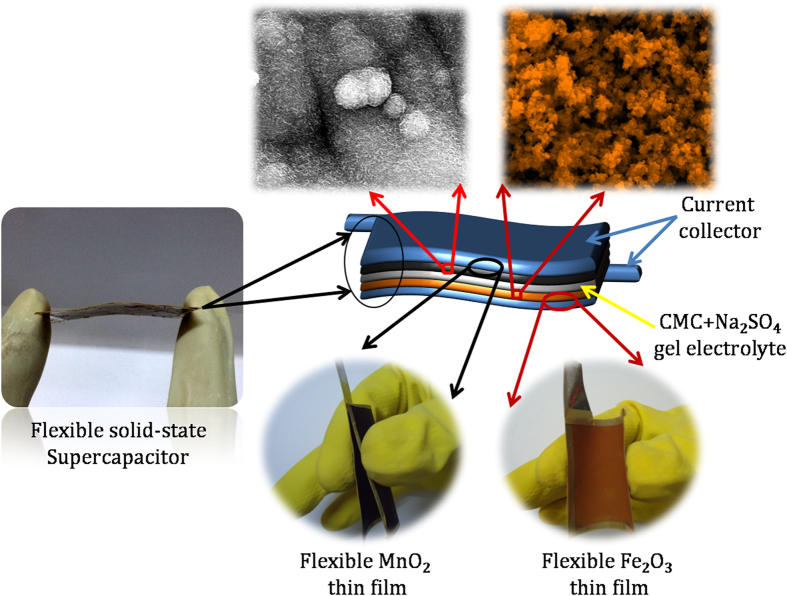
Schematic presentation of the fabricated asymmetric SC device based on flexible MnO_2_ NSs as positive electrode and Fe_2_O_3_ NPs as negative electrode with 1 M Na_2_SO_4_/CMC gel as separator and electrolyte.

The FSS symmetric MnO_2_//MnO_2_ and asymmetric MnO_2_//Fe_2_O_3_ supercapacitors were fabricated employing the neutral 1 M Na_2_SO_4_/CMC as a separator and gel electrolyte, and optimized MnO_2_ (electrodeposited) and Fe_2_O_3_ (SILAR deposited) electrodes, as illustrated in Fig. 4. All the electrochemical measurements were carried out at ambient conditions.

**Figure 5:**
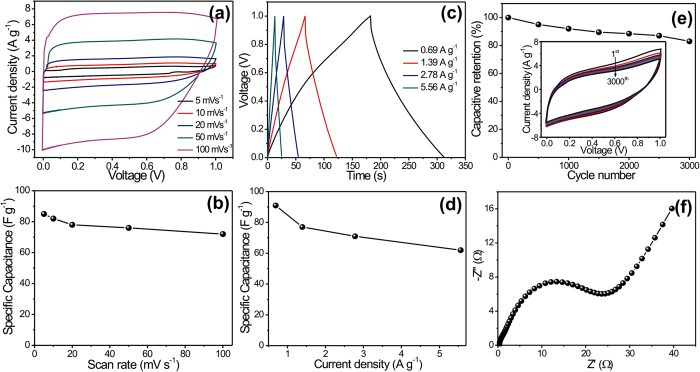
(**a**) CV curves and (**b**) specific capacitance at different scan rates, (**c**) GCD profile and (**d**) specific capacitance at various current densities, (**e**) capacitive retention as a function of cycle number; inset is the CV curves at different CV cycles and (**f**) Nyquist plot of MnO_2_/MnO_2_ symmetric FSS-SC device.

The CV measurements of symmetric FSS-SC device (MnO_2_/MnO_2_) were carried out between 0 to +1 V at various scan rates; see Fig. 5(a). The specific capacitances of the device were evaluated in accordance with the CV examinations at different scan rates, and are plotted in Fig. 5(b). The maximum specific capacitance of 85 F g^−1^ was obtained at a scan rate of 5 mV s^−1^. As it could be expected, the specific capacitance diminishes gradually with increasing scan rate but only to a minor extent. The excellent capacitive behavior with the exceptional reversibility of symmetric FSS-SC device clearly reflects from the mirror-image of current response on reverse voltage in CV profile. Additionally, the galvanostatic charge–discharge (GCD) technique is a direct tool to examine the applicability of fabricated SC device, since this technique can easily evaluate the rate capability of a device by judging the rate of change of voltage with time during charging and discharging at various current densities within a stable potential window. The GCD curves of the symmetric FSS-SC device at different current densities are plotted, as a potential−time profile, in Fig. 5(c). The estimated values of specific capacitance at different current densities are plotted in Fig. 5(d), and show a maximum capacitance of 91 F g^−1^ at a current density of 0.69 A g^−1^. The observed difference in specific capacitances evaluated through GCD and CV measurement can be easily explained. Thus, the estimated specific capacitance through CV measurement is at a particular voltage, whereas GCD measurement furnishes an average capacitance over the voltage range of 0–1  V^43^,^44^. Furthermore, the long cycling (for 3000 CV cycles at 100 mV s^−1^ of scan rate) and electrochemical impendence measurements (with 10 mV of AC amplitude and within the frequency range of 100 mHz to 100 kHz) have verified the excellent cycling stability (83% of capacitive retention, see Fig. 5(e)) and ion transports (R_s_ = 0.3 Ω cm^−2^ and R_ct_ = 9.51 Ω cm^−2^, see the Fig. 5(f)) of fabricated symmetric FSS-SC device, respectively.

**Figure 6:**
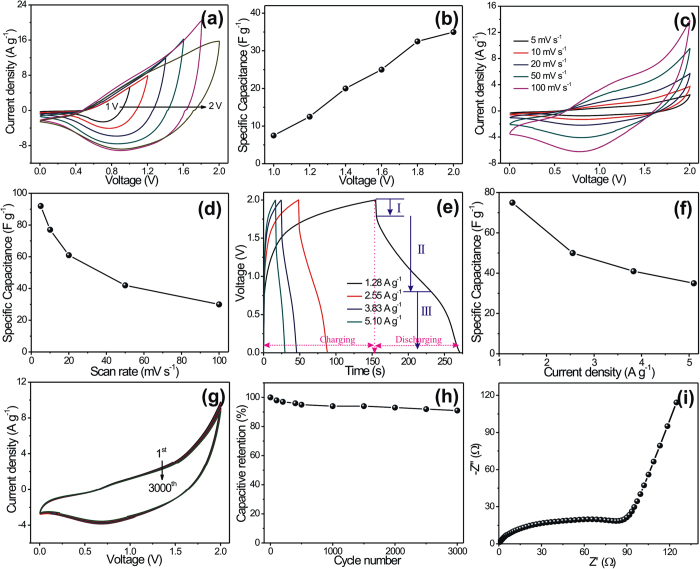
(**a**) CV curves and (**b**) specific capacitance as a function of voltage window, (**c**) CV curves and (**d**) specific capacitance at various scan rates, (**e**) GCD curves and (**f**) specific capacitance as a function of current densities, (**g**) CV curves and (**h**) capacitive retention at different cycle number, (**i**) The plot of the impedance analysis for MnO_2_//Fe_2_O_3_ asymmetric FSS-SC device.

The performance of an asymmetric FSS-SC device has also been investigated and is summarized in Fig. 6. Initially, an operating voltage from 1 to 2 V at 100 mV s^−1^ scan rate was used for the asymmetric (MnO_2_//Fe_2_O_3_) SC in accordance with CV studies of single electrodes, since the reversibility is the principal aspect affecting the power feature of SCs. The departure of CV curves from the ideal rectangular shape is associated with progression of faradic reactions at the surface of SC electrodes; see Fig. 6(a). The calculated values of specific capacitance are displayed in Fig. 6(b) as a function of operating voltage window. The gradual enhancement of specific capacitance for wide operating voltage window is clearly exhibited in Fig. 6(b). The reversibility was clearly apparent in CV profile for operating voltages up to 2 V, so the subsequent supercapacitive measurements of asymmetric FSS-SC device were investigated in 2 V of operating voltage window. Figure 6(c) displays, the CV curves at various scan rates from 5 to 100 mV s^−1^. The gradual fall of specific capacitance with increasing scan rate is clearly apparent from Fig. 6(d), which is the consequence of diffusion limits in charge transport at higher scan rates. The maximum specific capacitance of 92 F g^−1^ was obtained at 5 mV s^−1^ scan rate. The GCD measurements at various current densities were carried out and are shown in Fig. 6(e). The GCD curves of asymmetric FSS-SC device displays three variation region (see Fig. 6(e)) rapid drop in current at the starting of discharge is owing to internal resistance, (II) a linear deviation of the time dependence of the potential related to the double-layer capacitance behavior, and (III) slope variation of the time dependence of the potential represents a typical pseudo-capacitance behavior, which resulted from the electrochemical adsorption/desorption or redox reaction at an interface between electrode and electrolyte^45^. The values of specific capacitance for GCD study were calculated at different current densities and plotted in Fig. 6(f), where the maximum specific capacitance of 75 F g^−1^ was observed for a current density of 1.28 A g^−1^. The asymmetric FSS-SC device does not show the ideal shapes of CVs and charge-discharge curves typically found for EDLCs. Thus, CVs are not perfectly rectangular and charge-discharge curves are not linear. This is not surprising since oxides like MnO_2_ and Fe_2_O_3_ present pseudocapacitive behavior; which is just characterized by voltage-dependent charge transfer reactions, in which the charge used for the progression of an electrode process is a continuous function of potential^2^. Similar kind of results (i.e. redox process combined with EDLC i.e. adsorption/desorption of ions) for asymmetric supercapacitor have been widely reported in the literature^46^,^47^. Apart from this, the coulombic efficiency, stands for the efficiency of charge transfer during charging-discharging, and loss in coulombic efficiency as a function of current density were plotted in supporting information S6. The plot exhibits an improvement of coulombic efficiency (from 75.2 to 93%) with increasing current density. This may be consequence of easy deintercalation of Na ions from metal oxide lattice at high current density. An analogous response was observed in symmetric (MnO_2_ based) FSS-SC device as well as in previous reports^48^. This suggest at lower current density efficiency drops drastically as a consequence slow electrochemical reaction at nanostructured metal oxide electrodes and leads to irreversible reaction due to the stay of the device in critical potentials zone for a longer time. Additionally, the reversibility of asymmetric FSS-SC device have been studied at high current density for long term charging-discharging (3000^th^ cycle) measurements, see supporting information S6. The obtained coulombic efficiencies are 92.1 and 90.4% for 1^st^ and 3000^th^ cycles, respectively, which represents a very good reversibility of our asymmetric FSS-SC device at high current density. Moreover, the cycling stability of fabricated asymmetric FSS-SC device has been investigated for 3000 CV cycles at 100 mV s^−1^ scan rate; see the Fig. 6(g). The specific capacitance of the device decreased very slowly during the whole process and the capacitive retention after 3000 CV cycles was ca. 91%; as illustrated in Fig. 6(h). The excellent electrochemical stability of our asymmetric supercapacitor device as compared to previous studies (see the Supporting information S7) could be a consequence of: 1) low crystallinity of MnO_2_ and Fe_2_O_3_ electrodes leads to enhanced charge storage through adsorption/desorption of ions along with pseudocapacitive reaction, 2) conquer the limitation of dissolution of electrode material in aqueous electrolyte due to employment of solid state electrolyte and 3) the NPs Fe_2_O_3_ offers more surface area for charge storage together with vertically aligned NSs MnO_2_^49^,^50^,^51^. Figure 6(i) displays the Nyquist plot in the frequency range of 100 mHz to 100 KHz where the inclination of ca. 70° of the straight line followed by a semicircle denote excellent capacitive behavior. The values of R_s_ and R_ct_ obtained (0.64 and 28.95 Ω cm^−2^, respectively) indicate excellent ion transport through the asymmetric FSS-SC device fabricated.

## Discussion

**Figure 7:**
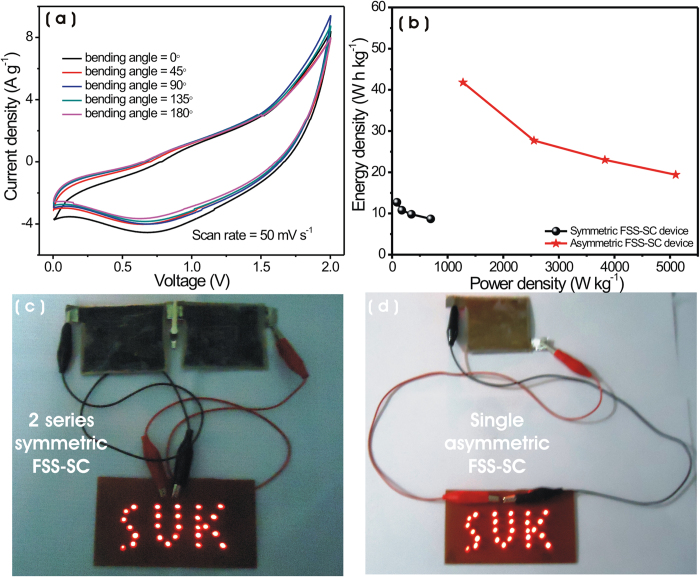
(**a**) CV curves of asymmetric FSS-SC device at different bent angles. (**b**) Plot of variation of energy density with power density of symmetric and asymmetric FSS-SC devices. Demonstration of (**c**) two symmetric FSS-SCs in series and (**d**) single asymmetric FSS-SC device light up significantly to SUK panel, which consists of 26 red LEDs in parallel arrangement and needs voltage more than 1.8 V.

In order to use our FSS-SC device for portable electronic application as a practical energy storage device, it should feature excellent mechanical flexibility along with high energy density and high power density. Accordingly, a series of CV measurements within 0–2 V window were carried out for various bending angles of 0°, 45°, 90°, 135°, and 180° at 100 mV s^−1^ scan rate (Fig. 7(a)). The mechanical bending of the device could cause a structural disintegration of the electrode, which could hinder its charge storage capacity^51^,^52^. However, the insignificant change in CV profile and admirable capacitive retention (94%) of the device even after 180° bending angle strongly supports the excellent flexibility and mechanical stability of the device (see supporting information S8). The bending test was executed consecutively and angles were altered manually, as indicated in inset of supporting Figure S8.

Furthermore, to investigate the efficiency of asymmetric FSS-SC device in energy storage capabilities in comparison with the symmetric device, we examined the energy density and power density at different current densities (for symmetric 0.69, 1.39, 2.78 and 5.56 A g^−1^, and for asymmetric 1.28, 2.55, 3.83 and 5.10 A g^−1^). The resulting energy densities and power densities for symmetric and asymmetric FSS-SC devices are displayed in Fig. 7(b). At a low power density, the symmetric SC has energy density of 12.7 Wh kg^−1^ (at 87 W kg^−1^), whereas asymmetric SC exhibits 41.8 Wh kg^−1^ of energy density (at 1276 W kg^−1^). While at high power density, the observed energy density for symmetric SC is 8.7 Wh kg^−1^ (at 694 W kg^−1^) and for asymmetric SC is 19.4 Wh kg^−1^ (at 5102 W kg^−1^). These results confirm that the asymmetric SC can offer a substantially boosted energy density (more than 2-fold) and power density as compared to the symmetric SC. A detailed literature survey of SC devices based on MnO_2_ and Fe_2_O_3_ electrodes is included in supporting information S7. The results and supercapacitive properties in the present study are superior to earlier reported values in various respects, namely, (1) the use of simple, cheap, additive-free, binder-less and environmentally friendly synthesis routes, which can deposit large area electrodes very fast, (2) use of cost-effective highly flexible stainless steel as a substrate, (3) the prepared electrode with the fine optimized nanostructures (NSs and NPS) exhibit the advantage of high flexibility (see Fig. 7 and supporting information S8), which is a necessary requirement for the development of flexible SCs and (4) the use of an inexpensive polymer gel of 1 M Na_2_SO_4_/CMC as a separator and electrolyte leading to an all-solid device. Accordingly, such a cost-effective asymmetric FSS-SC can be projected as energy storage device for high energy and high power applications. Finally, in order to check and confirm the improvement of energy density and power density, large area (4 × 5 cm^2^) FSS-SC devices (see the supporting information S9) were discharged through a panel displaying the achronim SUK (Shivaji University, Kolhapur), consisting of 26 red LEDs arranged in parallel, after being charged at 3 V. The panel was successfully turned on for two series combination of symmetric FSS-SC devices (see Fig. 7(C)), while it was lit effectively for single asymmetric FSS-SC device (see Fig. 7(d)). This experiment further proves that the asymmetric FSS-SC device offers 2-fold the energy density than the symmetric FSS-SC device and holds a great potential to meet present demanding trends for portable energy storage devices like high-performance and high flexibility.

In summary, we have shown an integral approach towards the development of economical, eco-friendly and highly flexible supercapacitor devices with enhanced energy storage and power capabilities through gentle and simple routes for the fabrication of the electrodes integrated with an inexpensive polymer gel electrolyte (1 M Na_2_SO_4_/CMC). The devices were fabricated with chemically-engineered thin film electrodes featuring porous surfaces (fine nanostructures with high surface area and microporosity) and favorable structural characteristics (low crystallinity lowering the lattice energy and promoting fuller utilization of electrode material). The asymmetric SC device fabricated could effectively be applied as a sturdy and flexible energy storage device, easily integrated with portable electronics.

## Methods

### Preparation of MnO_2_ NSs on highly flexible stainless steel

MnO_2_ NSs were developed on washed and cleaned large area (8 × 7 cm^2^) flexible stainless steel sheet by potentiodynamic mode of electrodeposition. Briefly, 0.1 M KMnO_4_ (AR, 99%) and 0.1 M KNO_3_ (AR, 99%) has dissolved in 200 ml of double distilled water (DDW), followed by stirring for 1 hr to form pinkish and precipitate free solution. Potentiodynamic deposition of MnO_2_ with the three-electrode configuration was made on flexible stainless steel as working electrode, graphite container and saturated calomel electrode (SCE) as counter and reference electrodes, respectively from +0.5 to +1.5 V/SCE at different scan rates (50, 100 and 200 mV s^−1^) for 100 cycles, illustrated in supporting information S1. At last, MnO_2_ NSs substrate was removed, rinsed in DDW, and dried at 353 K for 5 h.

### Preparation of Fe_2_O_3_ NPs on highly flexible stainless steel

Fe_2_O_3_ NPs have been prepared on pre-cleaned highly flexible stainless steel (8 × 7 cm^2^) sheet through the alternate immersion of sheet into iron sulfate (AR, 99%) bath (cationic source of Fe^2+^ ions) at room temperature and sodium hydroxide (AR, 97%) solution maintained at different reaction temperature (313, 333 and 353 K) (anionic source of OH^−^ ions). A four-beaker SILAR system was used to synthesis Fe_2_O_3_ thin film onto highly flexible stainless steel sheet. In brief, aqueous 0.05 M FeSO_4_ and 0.1 M NaOH solutions were used as a cationic (first beaker) and anionic sources, respectively. Foremost, cleaned flexible stainless steel sheet was immersed in the iron sulfate solution for 10 s to adsorb Fe^2+^ ions onto the substrate surface and succeeded through 10 s rinsing in DDW to take away the loosely bound species of Fe^2+^. Subsequent immersion of the substrate into the NaOH solution for 10 s, kept at different reaction temperature (313, 333 and 353 K) with the intention to optimize the smooth, uniform thin film with fine nanostructure, a reaction processed on the substrate surface to form Fe_2_O_3_ nanoparticles. Lastly, the substrates were rinsed in DDW to remove the excess or unreacted species. In this way, single SILAR cycle of Fe_2_O_3_ deposition was accomplished and 100 such deposition cycles were repeated to optimize the terminal thickness of film. Finally, prepared Fe_2_O_3_ NPs substrate was removed, rinsed in DDW, and dried at 353 K for 5 h.

### Fabrication of FSS-SCs

To fabricate the symmetric MnO_2_/MnO_2_ FSS-SC, MnO_2_ NSs (4 × 5 cm^2^) thin films were used as positive and negative electrodes, while to construct asymmetric MnO_2_//Fe_2_O_3_ FSS-SC MnO_2_ NSs and Fe_2_O_3_ NPs thin films with the same area were employed as positive and negative electrodes, respectively. The SC was typically compiled of two thin film electrodes separated by 1 M Na_2_SO_4_-carboxymethyl cellulose gel electrolyte, as illustrated in scheme 1. The detailed fabrication process was explained in supporting information S9.

### Characterization

The surface morphologies and structure of the electrode materials were characterized through field-emission scanning electron microscope (FE-SEM, JEOL JSM 6390), the X-ray diffraction (XRD) (Bruker axs D8, λ = 1.54 A°) and micro-Raman spectroscopy (Jobin Yvon Horibra LABRAM-HR visible spectrometer with an argon-ion continuous-wave laser (488 nm) as the excitation source). The volumetric N_2_ adsorption/desorption studies were executed, to investigate the surface area and pore structure of the electrode materials, by Brunauer-Emmett-Teller (BET) measurement using an ASAP-2010 surface area analyzer.

Cyclic voltammograms (CVs), galvanostatic charge-discharge (GCD) and electrochemical impedance measurements were examined at room temperature, to assess the electrochemical features of the electrodes and fabricated FSS-SC devices, using the electrochemical workstation (ZIVE SP5). The electrochemical impendence measurements were assessed in the frequency range of 100 kHz to 100 mHz with the AC amplitude of 10 mV. The electrochemical performance of MnO_2_ NSs and Fe_2_O_3_ NPs electrodes were analyzed through three-electrode system where thin film electrode as a working electrode, platinum as a counter electrode and saturated calomel electrode (SCE) as a reference electrode. The specific capacitance from the CV plot was computed through the following equation:


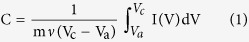


where C is the specific capacitance (F g^−1^), v is the potential scan rate (mV s^−1^), V_c_ − V_a_ is the potential window, I (V) denotes the response current (mA) and m is the mass of material on electrode for unit area (here 1 cm^2^), which is estimated through the following relation:


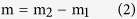


where, m_2_ is mass of the substrate with film and m_1_ is mass of the substrate without film. The specific capacitance (C_s_) from the potential–time profile has been calculated by the following equation:


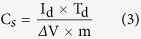


where I_d_ (mA) is the constant discharge current density (mA cm^−2^), T_d_ is the discharge time and ΔV (V) is the potential window.

The mass ratio of positive to negative electrodes was evaluated through using the following equation to maintain a charge balance q^+^ = q^−^ in order to fabricate the device


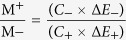


in which q^+^/q^−^, m^+^/m^−^, C^+^/C^−^, and E^+^/E^−^ are the charge, mass, specific capacitance, and potential windows for the cathode (+)/anode (−), respectively. Accordingly, the optimal positive-to-negative mass ratio was determined to be 0.85:1 for this asymmetric capacitor. Hence, the mass loadings of positive and negative electrodes were well balanced to 0.18 and 0.212 mg cm^−2^, respectively.

The energy density (E, W h kg^−1^) and power density (P, W kg^−1^) were estimated by following equations:


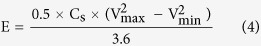



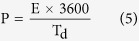


where, V is the potential drop during discharge i. e. ΔV (potential window).

## Additional Information

**How to cite this article**: Gund, G. S. *et al.* Low-cost flexible supercapacitors with high-energy density based on nanostructured MnO_2_ and Fe_2_O_3_ thin films directly fabricated onto stainless steel. *Sci. Rep.* **5**, 12454; doi: 10.1038/srep12454 (2015).

## Supplementary Material

Supporting Information
