# Supplementary material for: Low-cost flexible supercapacitors with high-energy density based on nanostructured MnO2 and Fe2O3 thin films directly fabricated onto stainless steel
Source: Sci Rep. 2015 Jul 24;5:12454. doi: 10.1038/srep12454 (PMC4513645; doi:10.1038/srep12454)
Supplement: Supporting Information [file srep12454-s1.pdf]

Supporting Information (SI)

Low-cost flexible supercapacitors with high-energy density based on nanostructured MnO_2 and Fe_2O_3 thin films directly fabricated onto stainless steel

Girish S. Gund[a,b,c], Deepak P. Dubal[c], Nilesh R. Chodankar[a], Jun Y. Cho[b], Pedro Gomez-Romero[c], Chan Park[b], Chandrakant D. Lokhande[a]***

[a]Thin Film Physics Laboratory, Department of Physics, Shivaji University, Kolhapur, - 416004 (M.S), India

[b]Department of Materials Science and Engineering, Seoul National University, Seoul 151-744, South Korea

[c]Catalan Institute of Nanoscience and Nanotechnology, CIN2, ICN2 (CSIC-ICN), Campus UAB, E-08193 Bellaterra (Barcelona), Spain

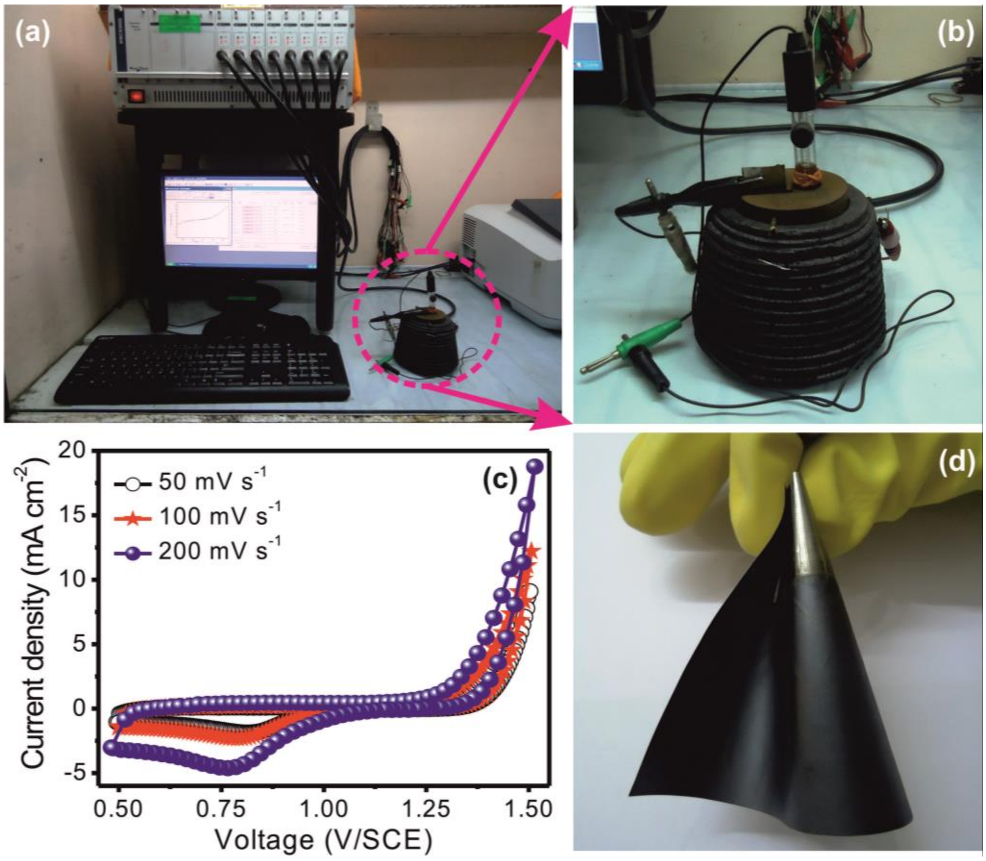

Supplementary Figure S1: Experimental setup and preparation of MnO_2 NSs electrodes. (a) The digital photograph of battery cycler with the modified cell constructed for the preparation of large area electrodes. (b) The magnified digital photograph of cell consisting graphite container as counter electrode, SCE as reference electrode, large area (8 × 7 cm^2) stainless steel sheet as a working electrode. (c) The CV curves during the deposition process, three curves of three electrode preparations at three different scan rates (50, 100 and 200 mV s^{-1}) (symbolized as MnO50, MnO100 and MnO200, respectively). (d) The digital photograph of MnO_2 NSs thin film electrode deposited at 50 mV s^{-1} scan rate and additionally demonstrating the flexibility of prepared electrode.

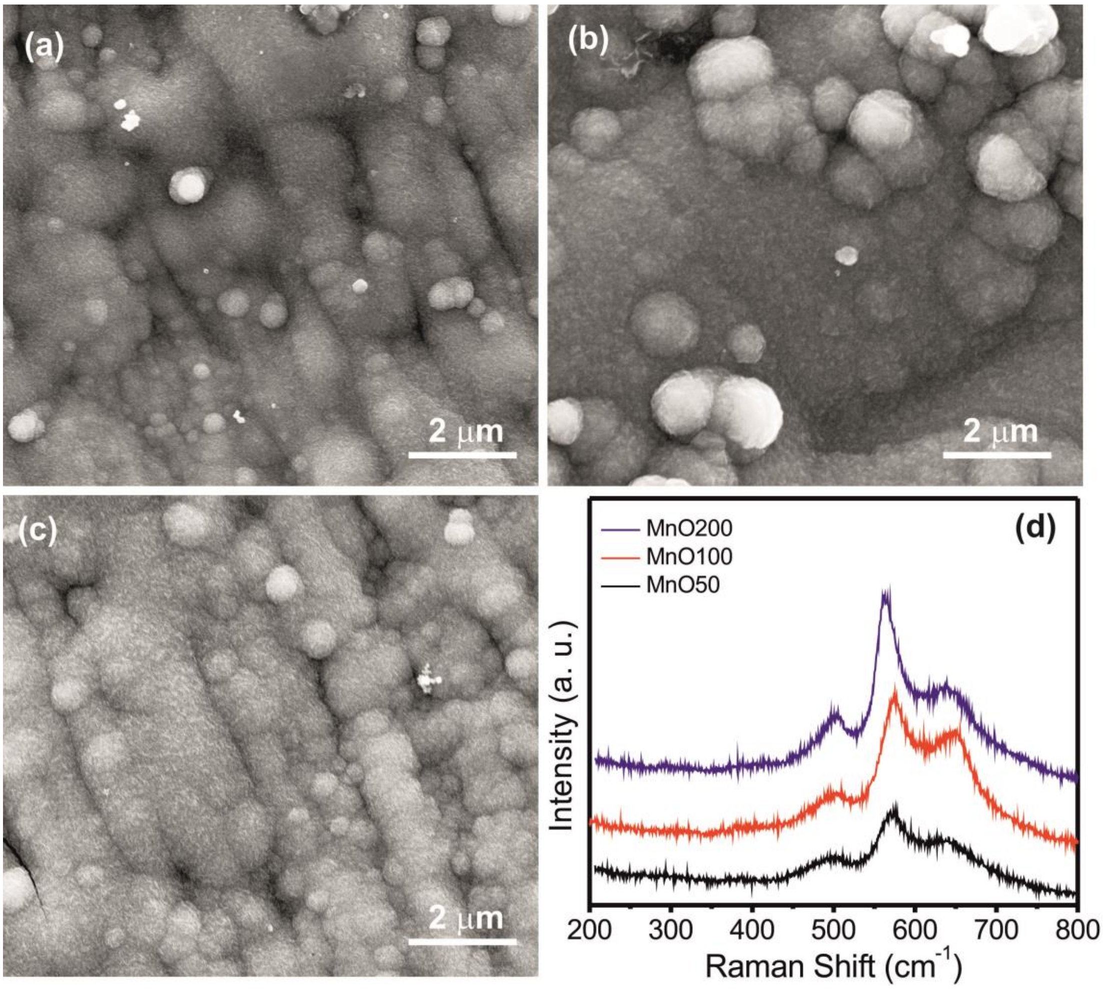

Supplementary Figure S2: The SEM image of MnO_2 electrodes prepared at three different scan rates of (a) 50, (b) 100 and (c) 200 mV s^{-1} using the potentiodynamic mode of electrodeposition. (d) Raman spectra of MnO50, MnO100 and MnO200. The SEM imagesof MnO500, MnO100 and MnO200 exhibits very smooth, fine and uniform surface morphology for MnO50 electrode, while other electrodes displays overgrowth of nanoclusters, which may be less electrochemical stable and distress charge storage capability on mechanical pressure. The less intensity of Raman peaks for MnO50 electrode is associated to its nanostructure and less crystallinity.

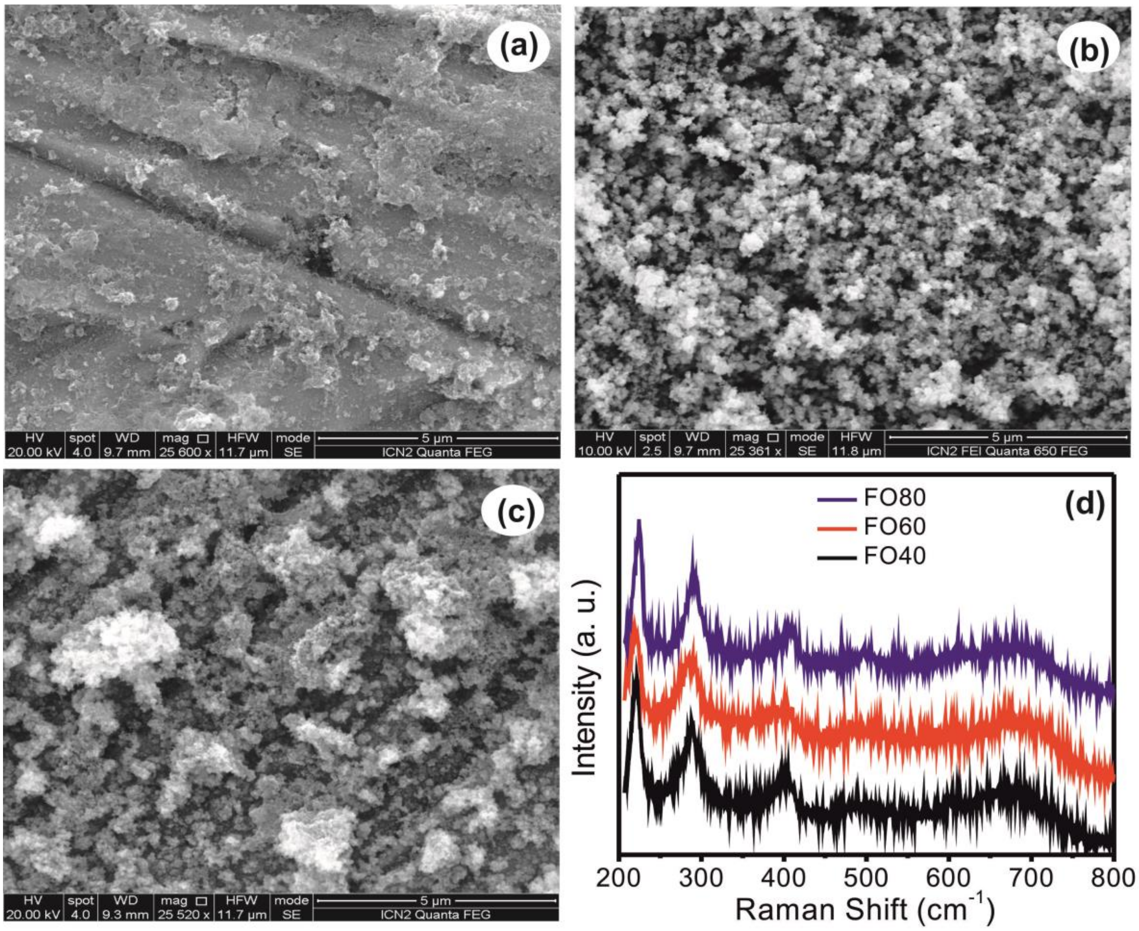

Supplementary Figure S3: The SEM micrograph of Fe_2O_3 electrodessynthesized through SILAR method at three different reaction temperatures (a) 313, (b) 333 and (c) 353 K, which furthermore symbolized as FO40, FO60 and FO80, used digits are associated to conversion of reaction temperature Kelvin to Celsius (d) Raman spectra of FO40, FO60 and FO80. The micrographs of three electrodes reflect the more uniformity of FO60 sample. In addition, the Raman spectroscopic examination of the three electrodes supports the outcomes of SEM analysis i. e. the nearly same height and broadness of Raman peaks for all samples are corresponds to insignificant variation in particle size.

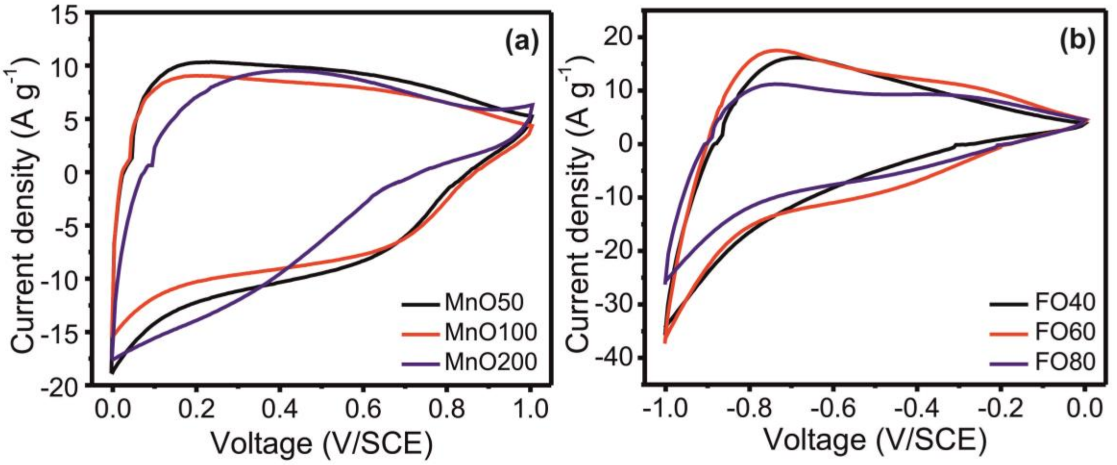

Supplementary Figure S4: The CV measurements of (a) MnO50, MnO100 and MnO200, and (b) FO40, FO60 and FO80 electrodes. The deviation of CV curves from rectangular shape is assorted to faradic reaction processing on electrodes. The high current density of MnO50 and FO60 electrodes reflect better charge storing capacity of respective electrodes.

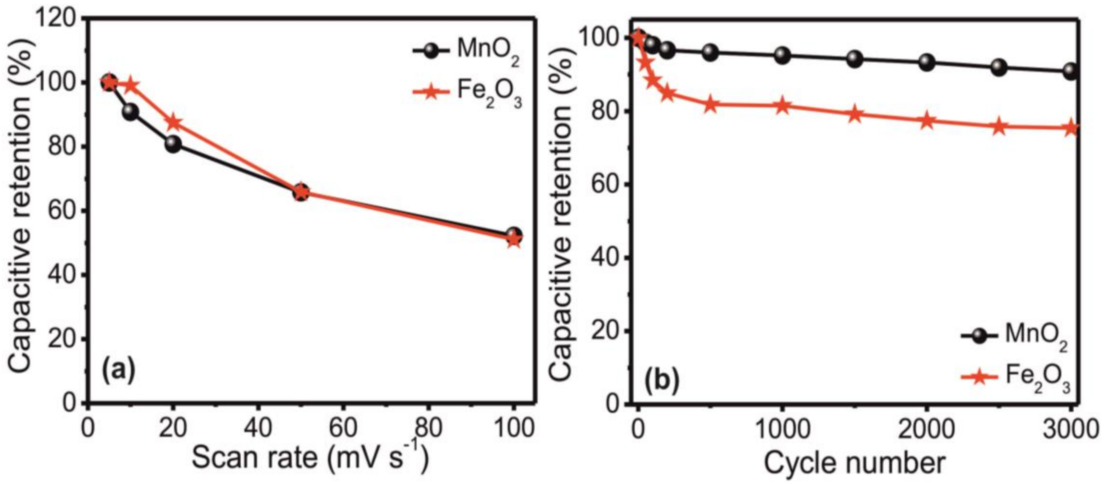

Supplementary Figure S5: The plot of capacitive retention with the (a) scan rate and (b) CV cycle numbers of MnO_2 NSs and Fe_2O_3 NPs electrodes. The decrement of capacitive retentions of both the electrodes with the increasing scan rate is linked to progression of redox reactions only on outer surface of electrodes due to inferior time at high scan rate. The plot of capacitive retention with the CV cycle number clearly reflects excellent electrochemical stability of MnO_2 NSs electrode as compare to Fe_2O_3 NPs electrode owing to more dissolution of electrode material in electrolyte with the cycling.

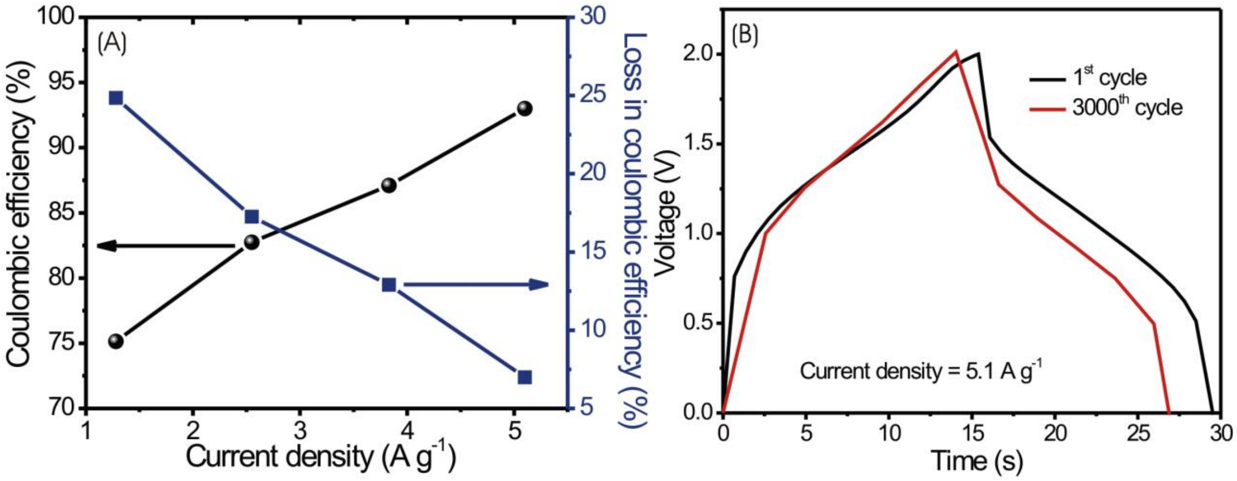

Supplementary Figure S6: (A) the coulombic efficiency and loss of coulombic efficiency as a function of current density, (B) the charge-discharge curves at different cycles.

Supporting information S7: Comparison of present asymmetric FSS-SC device with the earlier accounted asymmetric SC in terms of different aspects, like specific capacitance (C,), energy density (E,), power density (P,), stability (S,) and other components of SC device.

Electrode materials	Method/ Substrate	Electrolyte	C ($F g^{-1}$)	E ($Wh kg^{-1}$)	P ($W kg^{-1}$)	S (%)	Ref.
MnO_2// FeOOH	HT//HT (Powder)/ SS	Aqueous Li_2SO_4	116	12	3700	~85/2000	10
MnO_2// Fe_3O_4	SG//Ppt (Powder)/-	Aqueous K_2SO_4	20	7	820	-	11
MnO_2// Fe_2O_3	HT//STAH/ CF	Solid state LiCl/PVA	91.3	33.5	-	~82/6000	13
MnO_2// Fe_2O_3	PDE//SILAR/ SS	Solid state Na_2SO_4/ CMC	92	41.8	5102	91/3000	Present work
HT-hydrothermal, SG- Sol-gel, Ppt- Precipitation, STAH-sacrificial template-accelerated hydrolysis, PDE-potentiodynamic electrodeposition, SILAR-successive ionic layer of adsorption and reaction, SS-stainless steel, CF-carbon fabric							

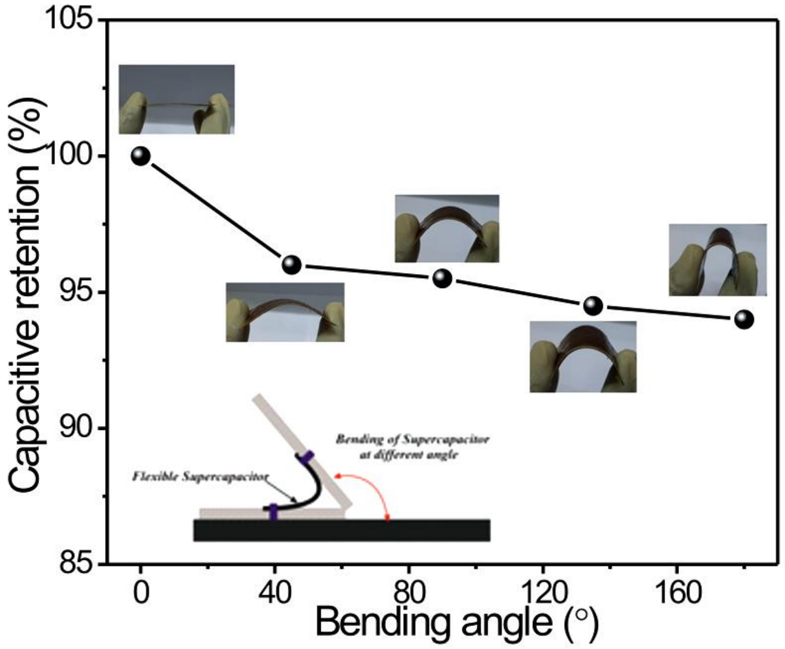

Supplementary Figure S8: Dependence of specific capacitance for asymmetric FSS-SC device on bent angle. Inset figures at the middle are digital photographs of asymmetric FSS-SC device at different bending angles, while at the bottom is schematic representation for flexibility test of device.

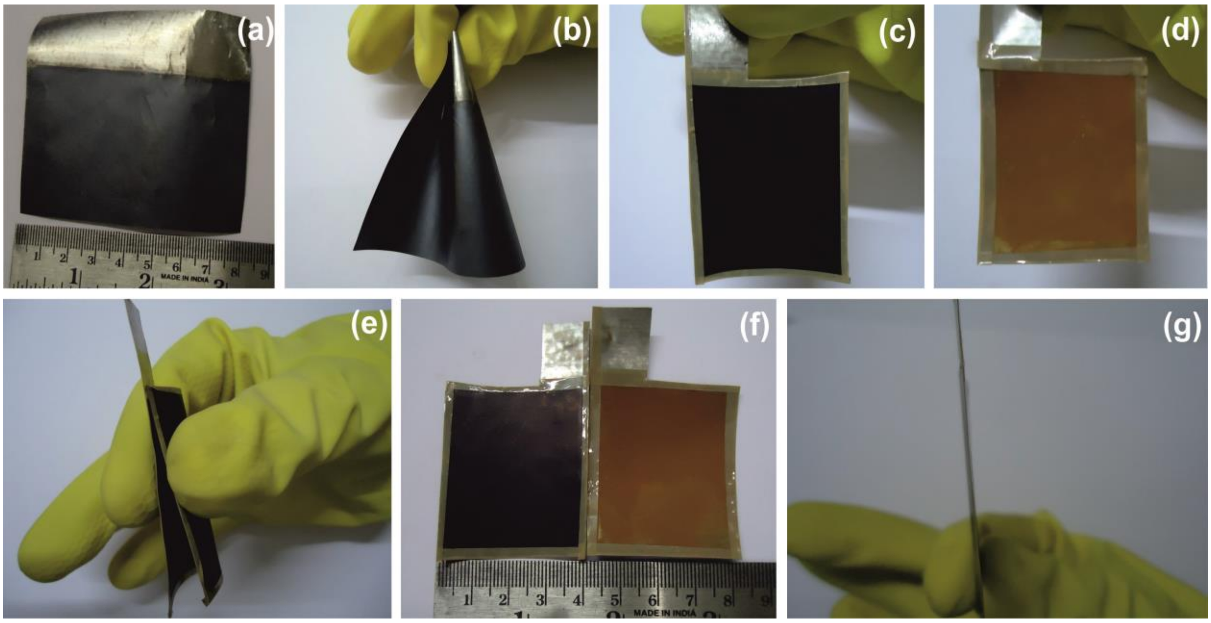

Supplementary Figure S9: Fabrication of symmetric and asymmetric FSS-SC devices. The digital photograph (a) of as prepared large area MnO_2 NSs electrode, (b) showing flexibility of electrode, (c) MnO_2 NSs and (d) Fe_2O_3 NPs electrodes with the sealed edges, (e) screening flexibility of sealed electrode, (f) presenting dimensions of electrode and (g) demonstrating slimness of prepared electrode.

Preparation of Gel Electrolyte

The gel electrolyte was composed through adding 3.0 g of Carboxymethyl cellulose (CMC) powder into 50 ml of double distilled, then solution is heated to 353 K with constant stirring till clear and viscous solution is obtained. Furthermore, 1 M Na_2SO_4 salt was added very slowly with constant stirring (without stirring or sudden addition of Na_2SO_4 leads crystallization of salt). This viscous solution is employed as gel electrolyte.

Fabrication of symmetric and asymmetric FSS-SC devices

The flexible stainless steelsubstrates deposited with MnO_2 NSs and Fe_2O_3 NPs were employed as electrodes to fabricate SC devices. The symmetric FSS-SC device was

constructed using MnO_2 NSs electrodes as positive and negative electrodes, whereas asymmetric FSS-SC device was made by employing MnO_2 NSs as a positive electrode and Fe_2O_3 NPs as a negative electrode. In order to construct devices, initially the edges of electrodes are sealed with adhesive tape to avoid any short circuit, see the supplementary Figure S7 (c) and (d). Consequently, 1 M Na_2SO_4/CMC gel electrolyte is painted on the electrodes and two electrodes are sandwiched in order to assemble supercapacitor. Furthermore, the pressure of about ~1 ton was enforced on the assembled SC device, in order to improve its mechanical features and contact. At the last, the device was kept in fume hood at room temperature to evaporate the excess water.
